# PSA change after antibiotic treatment should not affect decision-making on performing a prostate biopsy

**DOI:** 10.55730/1300-0144.5571

**Published:** 2022-12-23

**Authors:** Yunus KAYALI, Mevlana Derya BALBAY, Abdullah İLKTAÇ, Cevper ERSÖZ, Hüseyin TOPRAK, Kayhan TARIM, Arzu BAYGÜL, Muzaffer AKÇAY, Bayram DOĞAN

**Affiliations:** 1 Department of Urology, Sakarya Yenikent State Hospital, Sakarya, Turkey; 2 Department of Urology, Faculty of Medicine, Koç University, İstanbul, Turkey; 3 Department of Urology, Faculty of Medicine, Bezmialem Vakıf University, İstanbul, Turkey; 4 Department of Radiology, Faculty of Medicine, Bezmialem Vakıf University, İstanbul, Turkey; 5 Department of Biostatistics, Faculty of Medicine, Koç University, İstanbul, Turkey

**Keywords:** Antibacterial agents, multiparametric magnetic resonance imaging, prostate cancer, prostate-specific antigen

## Abstract

**Background/aim:**

To investigate the effect of antibiotic treatment on PSA when deciding on prostate biopsy.

**Materials and methods:**

A total of 206 patients with an elevated PSA level (2.5–30) were included. Mp-MRI could be done on 129 patients. Patients were given ciprofloxacin (500 mg, b.i.d. p.o.) for 4 weeks and PSA measurements were repeated. Systematic prostate biopsy was performed regardless of PSA changes on all patients. Additionally, cognitive biopsies were performed from PI-RADs III, IV, and V lesions.

**Results:**

Prostate cancer was detected in 36.4% of patients. 53.3% had Gleason score of 3+3, 46.7% had Gleason score ≥ 3+4. PSA values decreased in 56.3% and in 43.7% and remained the same or increased but cancer detection rates were not different: 34.5% vs. 38.9%, respectively (p = 0.514). PSA change in whole group was significant (6.38 ng/mL vs. 5.95 ng/mL, respectively (p = 0.01). No significant PSA decrease was observed in cancer patients (7.1 ng/mL vs. 7.05 ng/mL, p = 0.09), whereas PSA decrease was significant in patients with benign pathology (6.1 ng/mL vs. 5.5 ng/mL, p = 0.01). In patients with PI-RADs IV–V lesions, adenocarcinoma was present in 33.9% and 30.4% with or without PSA decrease, respectively (p = 0.209). Clinically significant cancer was higher in patients with after antibiotherapy PSA values >4 ng/mL regardless of PI-RADs grouping (p = 0.08). Addition of any PSA value to PI-RADs grouping did not have any significant effect on the detection of cancer.

**Conclusion:**

PSA change after antibiotic treatment has no effect in detecting cancer and should not delay performing a biopsy.

## 1. Introduction

Prostate cancer (PCa) is the most common cancer in male population in developed countries [1]. Serum prostate specific antigen (PSA) measurement has been used in PCa screening [2]. PSA can be easily affected by noncancer prostatic diseases and interventions to the prostate; therefore, it may show variations independent from PCa [3,4]. Measurements such as PSA velocity, PSA density (PSAD), age adjusted PSA are used to overcome the insufficiency of PSA in detecting prostate cancer [5,6]. Prostate biopsy is used to diagnose or rule out prostate cancer in patients with elevated serum PSA. Recently, MRI has been widely used for deciding whether a biopsy is indicated or not.

Since prostate infections are also observed among the causes of PSA elevation, it is thought that the probability of prostate cancer is lower in PSA levels that decrease after antibiotics. For this reason, there is an opinion that prostate biopsy should not be performed in PSA levels that tend to decrease. In this way, it is tried to prevent unnecessary biopsies.

We have investigated the value of antibiotic treatment and PSA change after such patients on deciding prostate biopsy. We also tried to correlate PSA kinetics after antibiotic treatment with the diagnosis of prostate cancer.

## 2. Materials and methods

This study was carried out at Bezmialem Vakıf University Medical School Hospital Urology Department with the approval of Bezmialem Vakıf University Medical School Hospital Ethics Committee dated 11.04.2018 and numbered 5830.

Following the approval of the local ethics committee 441 patients who applied to the urology outpatient clinic of Bezmialem Vakıf University Medical School Hospital between January 2017 and January 2018 and received antibiotic treatment due to high serum PSA level (2.5–30 ng/mL) and then underwent TRUS-Bx were included in this study.

Patients excluded from this study were those;

1. With suppressed immune system, severe coagulopathy, and those who received treatment for acute prostatitis,
2. Receiving 5-alpha reductase inhibitor therapy due to benign prostatic hyperplasia,
3. With a history of cystoscopy/TUR-Prostatectomy/TUR-Bladder operation in the last 3 months,
4. With a history of chronic prostatitis,
5. With other urinary infections,
6. With recently included urolithiasis,
7. With urinary retention which needed urethral catheterization.

**Figure 1:**
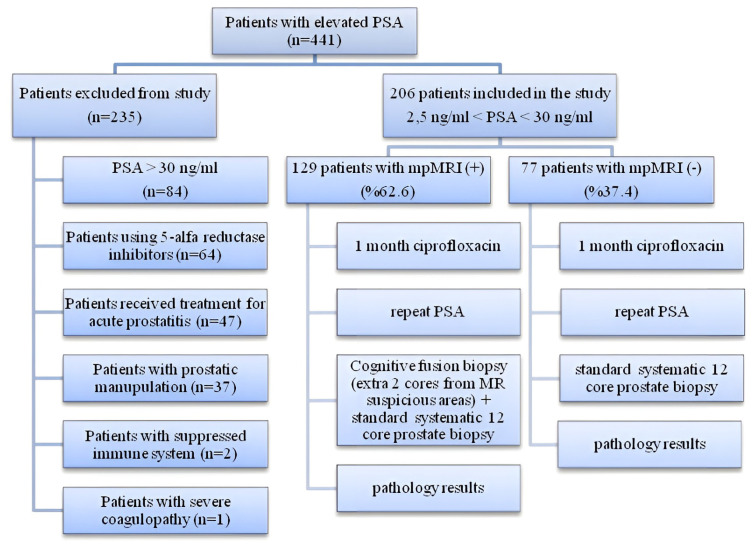
Patient selection.

Multiparametric magnetic resonance imaging (mpMRI) was recommended for all patients, but could not be done in 77 patients for various reasons (claustrophobia, cost, patient disagreement, etc.). Images of 129 patients who underwent mpMRI were evaluated by a single experienced radiologist and classified across PI-RADs v2. All patients were given ciprofloxacin (500 mg, twice a day p.o.) treatment for 4 weeks and PSA levels were repeated (PSAab). Prostate biopsy was performed regardless of PSA changes on all patients under local anesthesia and transrectal ultrasound guidance. A standard systematic 12-core biopsy was performed on 77 patients who did not undergo MRI. On 129 patients who underwent mp-MRI, cognitive biopsies were performed from PI-RADs v2 III, IV, and V lesions in addition to standard systematic biopsy as shown in Figure 1. Patient selection.

Demographic data such as age, mpMRI reports, digital rectal examination (DRE) findings, pretreatment PSA levels (initial PSA = PSAi), PSA values checked after treatment (PSAab) prostate volumes calculated on transrectal ultrasound examination and pathology results were recorded. Analysis and comparisons were performed retrospectively.

Statistical analyses were performed by using IBM Corp. Released 2019. IBM SPSS Statistics for Windows, Version 26.0. Armonk, NY: IBM Corp and MedCalc Statistical Software version 19.1 (MedCalc Software bv, Ostend, Belgium; https://www.medcalc.org; 2019). Mean and standard deviation were used to describe normally distributed continuous data, whereas median and minimum-maximum values were used to describe continuous variables which are not normally distributed. Frequencies (n) and percentages (%) were used as descriptive statistics for categorical variables. Normal distribution was evaluated with the Kolmogorov-Smirnov test. For the comparison of proportions chi-square test was used (or Fisher exact test where available). In order to compare two dependent, nonnormally distributed variables Wilcoxon Signed Rank test was used. Statistical significance was set as p < 0.05. As a multivariate analysis to evaluate the risk factors on malignant pathology logistic regression model was used. Lastly, a mathematical model was tried to be developed in order to find out the best model to predict both prostate cancer and significant prostate cancer by using the logistic regression function. The performance at diagnosing of this model has been inspected with receiver operating curve (ROC) analysis.

PSA changes before and after antibiotic treatment were evaluated separately for the following patient groups using the Wilcoxon signed-rank test:

- All patients,
- Patients with and without prostate cancer on biopsy,
- Patients with PI-RADS II–IV lesions on mp-MRI,
- Patients diagnosed with benign pathology and PCa for each PI-RADs group.

For the following parameters rates of benign pathology and prostate cancer with Gleason 3+3 or higher were evaluated using the chi-square test or Fisher’s exact test:

- Patient age,
- Initial PSA,
- PSA density,
- PSA change (PSAab ≤ 4.0ng/mL vs. PSAab > 4.0ng/mL, PSA decrease ≤ %50 vs. > % 50),
- DRE findings,
- PI-RADs groups.

Benign pathology and PCa rates according to PSA changes as well as Gleason score 3 + 3 and higher-grade cancer rates in patients with PCa were evaluated according to the following parameters and compared using the chi-square test or Fisher’s exact test:

- Patient age,
- Initial PSA,
- PSA density,
- PSA change (PSAab ≤ 4.0ng/mL vs. PSAab > 4.0ng/mL, PSA decrease ≤ %50 vs. > %50),
- DRE findings,
- PI-RADs groups.

Initial PSA and PSAab (PSA levels after 4 weeks antibiotherapy) were evaluated separately for the following patient groups using Wilcoxon signed-rank test for all patients, patients with and without PCa on biopsy, with PI-RADs II–IV lesions, with benign pathology and PCa for each PI-RADs group.

Significant PCa was defined as ≥ Gleason 3+4, or >3 biopsy cores positive, or at least one biopsy core with >50% involvement. Insignificant PCa was defined as ≤Gleason 3+3 without Gleason pattern 4 or 5 as tertiary pathology, and less than 3 core samples, and no core sample >50% involved.

Correlation of benign pathology and any PCa or significant PCa and the remaining patients were evaluated for patient age, PSAi (initial PSA), PSAD (density of PSA), PSA change (PSAab ≤ 4.0ng/mL vs. > 4.0ng/mL, PSA decrease ≤ %50 vs. >%50), DRE (digital rectal examination) findings, PI-RADs groups using chi-square test or Fisher’s exact test. Statistical significance was set as p < 0.05.

To evaluate the risk factors on malignant pathology multivariate analysis was done using the logistic regression model. A mathematical model was developed to predict both PCa and significant PCa by using the logistic regression function. Performance at diagnosing this model has been inspected with ROC (receiver operating curve) analysis.

## 3. Results

Demographic data of 206 patients participating in the study are shown in Table 1. The mean PSAi was 8.07 ± 5.15 ng/mL, and 59.7% of them were found to be between 4.1–10 ng/mL. Prostate adenocarcinoma was detected in 36.4% of patients after standard ± cognitive prostate biopsies performed in 206 patients with a high PSAi (>2.5 ng/mL) regardless of PSAab values (53.3% had a Gleason score of 3+3, and 46.7% had a Gleason score ≥ 3+4).

PSA values decreased in 56.3% and 43.7% of patients remained the same or increased in 4 weeks of antibiotic treatment. Median PSAi and median PSAab were 6.38 ng/mL (IR: 4.68–6.38) and 5.95 ng/mL (IR: 4.30–8.30), respectively (p = 0.01) as shown in Table 2.

No significant PSA decrease was observed with antibiotic treatment in patients with PCa (PSAi: 7.1 ng/mL, PSAab 7.05 ng/mL, p = 0.09), however, a statistically significant PSA decrease was evident in patients with benign pathology (PSAi: 6.1 ng/mL, PSAab: 5.5 ng/mL, p = 0.01). A minimal decrease in absolute PSA value was observed with antibiotic treatment in patients with PI-RADs IV lesions (PSAi: 6.76 ng/mL, PSAab 6.7 ng/mL, p = 0.033). Among patients with PI-RADs IV lesions only and patients with PI-RADs IV and V lesions but with benign pathology, a significant PSA change was observed (PSAi: 7.44 ng/mL, PSAab: 5.9 ng/mL, p = 0.002 and PSAi: 7.45 ng/mL, PSAab: 5.9 ng /mL, p = 0.02, respectively). As shown in Table 2, no statistically significant PSA decrease was observed with antibiotic treatment.

There was no difference in PCa detection rates between patient groups (PSAi: 2.5–4 ng/mL vs. 4.1–10 ng/mL vs. >10.1 ng/mL) (p = 0.830). The incidence of Gleason 3+3 cancer was higher if PSAi < 4.0 ng/mL compared to those with a PSAi >10 ng/mL (14.7% vs. 12%, p = 0.05) as shown in Table 3. Cancer detection rates were significantly higher in PI-RADs IV–V group (PI-RADs IV–V vs. II; p = 0.01, PI-RADs IV–V vs. III; p = 0.01).

Thirty out of 206 patients had suspicious findings on DRE, PCa was detected in 19 of 30 patients with suspicious DRE findings (7 Gleason score of 3+3 (Gleason group 1) and 12 Gleason score of 3+4 and higher (Gleason group 2+). Both PCa rates and clinically significant PCa rates were found to be higher in patients with suspicious DRE findings (p = 0.001, p = 0.0, respectively) as shown in Table 3. Likewise, incidence of PCa was higher in patients with both suspicious DRE findings and PSAab > 4 ng/mL (%63.3 vs. %31.8, p = 0.001).

The incidence of PCa increased parallel to PI-RADs score. When patients were divided into two groups as PI-RADs II and III–IV, incidences of PCa and clinically significant PCa were higher in patients with PI-RADs III+IV+V lesions (13.6% vs. 50.6%, p = 0.001 and 16.7% vs. 72.1%, p = 0.015). Accordingly, when patients were divided into two groups as PI-RADs II+III and PI-RADs IV+V, incidences of PCa and clinically significant PCa were similarly higher in patients with PI-RADs IV+V lesions (17.8% vs. 64.3%, p = 0.001 and 30.8% vs. 77.8%, p = 0.005).

No correlation was found between PSA change and PCa diagnosis in PI-RADs II and III groups as shown in Table 2. A statistically significant difference in PSAab was seen in patients in PI-RADs IV group. Median PSAi value of 45 patients in PI-RADs IV group 6.76 ng/mL (IR: 4.75–9.85) went down to 6.70 ng/mL (IR: 4.58–8.10) (p = 0.033). No significant difference was found in PSAi and PSAab when PI-RADs IV and V groups were combined. Median PSAi value of 56 patients in PI-RADs IV + V group was 7.2 ng/mL (IR: 4.9–10.12) which went down to 6.95 ng/mL (IR: 5.05–8.3) (p = 0.2). We did not see any significant PSA change with antibiotic treatment in either PI-RADs IV or IV + V groups if the patient had PCa as shown in Table 2. In patients with benign pathology and PI-RADs IV lesion median PSAi was 7.44 ng/mL (IR: 4.95–14.11) which went down to 5.9 ng/mL (IR: 4.53–8.2) with antibiotic treatment (p = 0.002). There was no patient with benign pathology in PI-RADs V group as shown in Table 2.

In patients with PI-RADs II lesions; PCa was detected in 6.8% of patients whose PSA decreased and 6.8% of patients whose PSA remained the same or increased. In patients with PI-RADs III lesions and PSA decrease after antibiotic treatment, PCa was detected in 17.2%. In cases with PI-RADs IV–V lesions and PSA decrease after antibiotic treatment, PCa was detected in 33.9% vs. 30.4% of cases whose PSA remained the same or increased. In patients evaluated with mp-MRI, clinically significant PCa rates were higher if both PSAi and PSAab were >4 ng/mL regardless of PI-RADs grouping (p = 0.080). PSAD values were significantly different between patients with PCa and benign pathology (PSAD ≤ 0.1 p = 0.039, PSAD p = 0.15 p = 0.041, PSAD ≤ 0.2 p = 0.003). However only PSAD ≤ 0.1 was able to predict clinically significant PCa (p = 0.001).

**Figure2:**
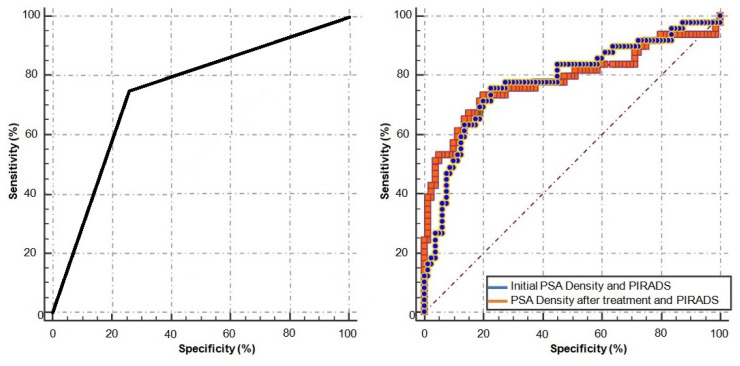
ROC Curves. The curve on the left showing the relationship between presence of PI-RAD-s IV+V lesions and prostate cancer. The AUC was 0.742 (p < 0.0001). On the right, the curve showing the relationship between the initial PSA density and the PIRADs score was shown in blue and the AUC was 0.782 (p < 0.0001). The curve showing the relationship between PSA density after 4 weeks of antibiotic treatment and PIRADs score was shown in orange and the AUC was 0.780 (p < 0.0001). There was no statistical difference in the comparison of the two curves with each other (p: 0.9247). (AUC = area under the curve).

Performance of PI-RADs IV+V lesions in predicting PCa was slightly but not significantly higher when PSAD both before and after antibiotic treatment were included in the analysis (AUC: PI-RADs IV+V = 0.742, initial PSAD and PI-RADs IV+V = 0.782, posttreatment PSAD and PI-RADs IV+V = 0.780, p = 0.924) as shown in Figure 2: ROC Curves.

Lastly, to eliminate the effect of insignificant PCa, we grouped our patients as those with a diagnosis of significant PCa (Gleason Score > 3+3) and the remainder of patients (either with an insignificant PCa as a Gleason 3+3 = 6 cancer and those with a benign histology). In patients with an MRI, patients with a significant PCa have a higher mean PSAi (8.47 ng/mL vs. 7.49 ng/mL, p = 0.033), less common unremarkable digital rectal examination findings (20.9% vs. 79.1%, p = 0.014), a higher PSAD (p = 0.001 and p = 0.005), and a lower rate of PI-RADs 2 and PI-RADs 2+3 lesions (2.3% vs. 97.7%, p = 0.001 and 5.5% vs. 94.5% p = 0.001, respectively). When all patients are included and MRI findings are ignored, mean age of patients with significant PCa was 65.21 (vs. 62.21), are older than 60. These patients also have a higher PSAi (9.31 ng/mL vs. 7.65 ng/mL, p = 0.036), higher PSAab 8.68 ng/mL vs. 6.31 ng/mL, p = 0.001, higher incidence of suspicious digital rectal examination findings (56.7% vs. 43.3%, p = 0.001) and a higher PSAD (p = 0.001 and p = 0.002, except for PSAD = 0.15).

## 4. Discussion

Although it has been used for prostate cancer detection since its first day of clinical use, PSA is an organ-specific not a disease specific marker and it can be measured high in all diseases (acute or chronic prostatitis, urinary retention) or interventions (prostatic manipulations, urinary catheterization) affecting the prostate. It is also known that PSA levels may increase parallel to the increasing prostate volume [5]. Besides, as shown in many studies PSA levels increase with age [6,7]. Upper limit of serum PSA was used to be accepted as 4.0 ng/mL, however recent studies showed that in 20% of patients with prostate cancer PSA values are below 4.0 ng/mL and therefore currently the upper limit value is considered as 2.5 ng/mL [8]. In addition to all these, there are also studies that reveal the fluctuating course in PSA levels, especially in recent years [9].

DRE findings and PSA levels play an important role in the decision making of performing prostate biopsy. Although these two markers are used together, they cannot be found basis to a sufficient sensitive and specific indication for systematic TRUS-Bx. Prostate cancer is detected in 34% of patients who undergo TRUS-Bx because of elevated PSA levels [9]. In other words, 66% of prostate biopsies are performed unnecessarily and some of these patients may experience complications after biopsy. Also, in up to 30% of patients the diagnosis of cancer can be missed with TRUS-Bx, and then repeated biopsies may be required [10]. Serefoglu et al. evaluated the success of 12-core prostate biopsy ex vivo and reported a false negative rate of over 30% [11]. In order to increase the yield of TRUS-Bx, additional parameters such as PSA density, total/free PSA ratio, PSA velocity [12,13], and PSA decrease after antibiotherapy [14] are used in the decision-making process. The sensitivity and specificity of TRUS-Bx have been reported to increase with mp-MRI, which is widely used recently.

The positive effect of antibiotic treatment on PSA reduction has been shown in many studies [14–16]. In studies on the clinical utility of this PSA decrease following antibiotic treatment in detecting prostate cancer, it is claimed that the risk of diagnosing prostate cancer is decreased if PSA reduces [14,17]. On the contrary, some studies showed that there is no relationship between PSA decrease after antibiotherapy and PCa detection on biopsy [18]. In the study performed by Bulbul et al., 48 patients with PSA levels between 5.0–28.5 ng/mL were evaluated and a PSA decrease was observed in 52% of the patients after 2 weeks of ciprofloxacin treatment [14]. Prostate cancer was detected in 9 out of 23 patients whose PSA level did not decrease. They recommended not to perform a biopsy in patients with decreased PSA after antibiotic treatment. As no biopsy was performed in patients with decreased PSA, it is not possible to make a pathological comparison between patients with and without PSA decrease. Atalay et al. evaluated 88 patients who were given ciprofloxacin treatment for 3 weeks and 89 patients who had no antibiotic treatment and performed TRUS-bx to all patients regardless of the final PSA value. While a PSA decrease was observed in 46.5% of patients in the antibiotic group, surprisingly a decrease in PSA was also observed in 15% of the patients who did not receive antibiotherapy (p = 0.022). Interestingly, in this study, PCa rates were reported to be higher in patients with PSA decrease after antibiotherapy (p = 0.011), but there was no difference in Gleason scores between groups [18]. Serretta et al. performed a study on 99 patients with PSA values between 4–10 ng/mL who received 3-weeks of ciprofloxacin treatment and found that the incidence of cancer in patients with increased PSA after antibiotherapy was higher than that of patients with a decreased PSA (40% vs. 20.3%, respectively, p = 0.02). The formation of a heterogeneous study group due to the low number of patients included and the differences in biopsy regimen (the number of biopsy cores taken ranged between 12–21 cores) are the limitations of their study [17]. In the current study, 206 patients who received antibiotic treatment for 4 weeks were evaluated, and a statistically significant decrease was observed in PSA levels after antibiotic treatment (PSA: 6.38 (IR: 4.68–9.8) ng/mL, PSAab: 5.95 (IR: 4.3–8.3) ng/mL, p = 0.001). Prostate biopsy was performed in all patients regardless of serum PSA change. All patients underwent a fairly similar prostate biopsy with all receiving systematic biopsy and cognitive fusion biopsy if MRI suggested a suspicious lesion. There was no statistically significant difference in cancer diagnosis rates between the groups with and without decreased PSA levels (34.5%, 38.9%, respectively, p = 0.514). This finding shows us that although PSA may decrease after antibiotherapy, PCa can still be detected in these patients at a similar rate.

Serretta et al. found in their study that the bigger the rate of PSA decline, the lower the diagnosis of PCa. They also reported that patients with PSA values returning to the normal range after antibiotherapy did not require biopsy [17]. Baltacı et al. found a 7.15% decrease in PSA levels at the end of 3 weeks of antibiotic treatment in their study on 100 patients with PSA values between 4–10 ng/mL. In their study 5 out of 17 patients (29.4%) with PSA value below 4 ng/mL were diagnosed with prostate cancer. Same group looked at the effect of antibiotic treatment on PSA change on prostate cancer detection rate. They found no significant difference in PSA levels between the patients with and without prostate cancer, but there was a decrease in free PSA values of patients with cancer. As a result, they reported that a decrease in PSA after antibiotic treatment would not eliminate the risk of prostate cancer [19]. In our study, prostate biopsy was performed in all patients and we did not see any significant difference in PSA levels in patient groups with and without prostate cancer (26.86 ± 23.84%, 23.52% ± 21.78, respectively, p > 0.05). PCa was found in 35.4% our patients whose PSA levels decreased below to 4.0 ng/mL after antibiotherapy. On the other hand, cancer detection rate was 40% in patients with PSA value remained above > 4.0 ng/mL, and there was no statistically significant difference between these two groups (p = 0.571).

In our study, prostate biopsy was performed on 129 (38%) patients who underwent mp-MRI and PCa was detected in 49 of these patients. PCa was detected in an increasing frequency parallel to PIRADS scoring: 13.6% of cases with PI-RADs II, in 24.1% of cases with PI-RADs III, in 55.5% of cases with PI-RADs IV, and in 64.3% of cases with PI-RADs IV+V, which is consistent with the literature. Park et al. reported the rate of prostate cancer detection as 17% in patients with PIRADs I and II lesions in their meta-analysis [22] which is comparable with our finding of a 16.3% rate of PCa in patients with PIRADs < III lesion. Unfortunately, since the number of patients with PIRADs III lesions in the presented study was small, no statistical analysis could be carried out to evaluate the presence of PCa in these patients.

To our knowledge, no correlation between PSA decline and PI-RADs scoring has been studied so far. Therefore, in order to evaluate the effect of PSA decrease in detecting PCa, we also looked at PCa rates according to PSA change in PI-RADs groups in the current study, in patients who underwent mp-MRI, PCa was observed in 38% (n = 27) of the patients with PSA decrease, and in 37.9% (n = 22) of the patients whose PSA did not change or increased. These results show that PCa is still detected at the same rate in patients with suspicious lesions in mp-MRI, regardless of the PSA change after antibiotic treatment (38% vs. 37.9%, p = 0.991).

Zhang et al. performed a study including 273 patients with PI-RADs < 3 and 218 patients with PI-RADs = 3 lesions and reported that low PSAD increased the negative predictive value in detecting overall PCa and clinically significant PCa [20]. Woshino et al. reported that patients with PI-RADs ≤ III and PSAD <0.15 do not need biopsy [21]. We have observed that combining any PSAD (>0.10, >0.15, >0.20) and PIRADs IV+V lesions were found to be effective in predicting PCa, but the presence of PIRADs IV+V lesions alone is more important evidence suggesting PCa than its combination with PSAD (AUC = 0.782, AUC = 0.742, respectively).

These findings suggest that the presence of PCa cannot be ruled out even if PSA decreases after antibiotic treatment in any PI-RADs score, in other words, PSA decrease does not affect the detection of prostate cancer, since prostate cancer is found at the same rate in patients with and without PSA decrease. This study, together with the literature data, supports the idea that prostate biopsy is not necessary in patients with PIRADs < III lesions.

In our study, a decrease was observed in PSA levels after antibiotic use, but no correlation was observed between the decrease and the incidence of prostate cancer. Therefore, the use of antibiotics to reduce high PSA levels in order to prevent unnecessary biopsies was not found significant and not recommended. In addition to all these, studies conducted in recent years reveal that long-term antibiotic usage (especially fluoroquinolone group) before prostate biopsy, increases the risk of sepsis caused by resistant microorganisms after biopsy [23].

As a conclusion, in daily urology practice, there may be a tendency not to perform prostate biopsy in a large number of patients with high PSA levels who have a decrease in PSA after antibiotic treatment. Results of this study showed that there is no significant difference in the diagnosis of cancer regardless of PSA decrease after antibiotherapy. This led us to think that a decrease in PSA level after antibiotic treatment could not rule out the presence of prostate cancer. Prostate biopsy should be performed in patients with PIRADs IV+V lesions without any antibiotic trial. In patients who do not have mp-MRI and PIRADs classification for any reason, deciding prostate biopsy should not be based on PSA change after antibiotic treatment and, we recommend that patients should be biopsied. Since sufficient information cannot be provided in patients with PIRADs III lesions with the available data, prostate biopsy should be performed in these patients as well. Studies involving a higher number of patients will be instructive to see the relationship between PSA change in patients with PIRADs III lesions after antibiotherapy and presence of PCa more clearly.

## Figures and Tables

**Table 1:** Patient demographics.

		n	Mean
Age		206	62.97 ± 7.68 (44–84)
PSA (ng/dL)	Initial	206	8.07 ± 5.15 (2.59–29.80)
	After treatment	206	6.90 ± 4.09 (1.00–27.00)
Prostate volume (cm^3^)		206	58.72 ± 28.99 (15.0–190.0)
PSA density		206	0.1670 ± 0.1445 (0.0316–1.0533)
		n	Percent (%)
mpMRI	Not performed	77	37.4%
	Performed	129	62.6%
PI-RADS	I	0	0%
	II	44	34.1%
	III	29	22.5%
	IV	45	34.9%
	V	11	8.5%
DRE	Not suspicious	176	85.4%
	Suspicious	30	14.6%
Pathology	Benign	131	63.6%
	Malignant	75	36.4%
Gleason Group 1	Gleason 3+3	40	53.3%
Gleason Group 2	Gleason > 3+3	35	46.7%

**Table 2:** Relationship of prostate cancer (PCa) and mp-MRI PI-RADs scores with the initial PSA and PSA after 4 weeks of antibiotic treatment.

	Before antibiotic treatment		After antibiotic treatment (4 wk)		
	PSAi (ng/mL) (median)	IR (…−…)	PSAab (ng/mL) (median)	IR (…−…)	p
All patients (n = 206)	6.38	4.68–9.8	5.95	4.3–8.3	0.001*
PCa (−) (n = 131)	6.1	4.53–9.0	5.5	4.3–7.9	0.010*
PCa (+) (n = 75)	7.1	4.8–10.16	7.05	4.2–8.6	0.097
PI-RADs II (n = 44)	5.86	4.06–9.98	5.34	4.14–8.98	0.329
PI-RADs III (n = 29)	5.8	3.82–7.5	4.4	3.14–6.27	0.183
PI-RADs IV (n = 45)	6.76	4.75–9.85	6.7	4.58–8.10	0.033*
PI-RADs V (n = 11)	7.59	7.12–10.2	7.68	6.2–10.5	0.306
PI-RADs IV+V (n = 56)	7.2	4.9–10.12	6.95	5.05–8.3	0.200
PCa (−) PI-RADs II (n = 38)	5.96	4.18–1.43	5.53	4.30–9.05	0.441
PCa (+) PI-RADs II (n = 6)	4.33	3.16–7.0	3.74	2.45–9.95	0.600
PCa (−) PI-RADs III (n = 22)	5.78	3.30–7.0	4.55	3.35–4.22	0.414
PCa (+) PI-RADs III (n = 7)	6.1	3.94–9.30	3.9	1.7–6.4	0.204
PCa (−) PI-RADs IV (n = 20)	7.44	4.95–14.11	5.9	4.53–8.2	0.002*
PCa (+) PI-RADs IV (n = 25)	6.3	4.34–9.15	7.2	4.7–8.05	0.773
PCa (−) PI-RADs IV+V (n = 20)	7.44	4.95–14.11	5.9	4.53–8.2	0.020*
PCa (+) PI-RADs IV+V (n = 36)	7.16	4.83–9.68	7.2	5.38–8.32	0.462

* Wilcoxon signed rank test

**Table 3:** Relationship of patient age groups, initial PSA, PSA density, PSA decline pattern, DRE findings, mp-MRI findings with prostate cancer and Gleason groups. (Gleason group 1: Gleason 3+3, Gleason group 2: Gleason 3+4, and higher).

Parameter		Benign	Prostate Ca	Gleason Group	
				I (%)	II+ (%)
Patient Age	44–60	52 (72.2%)	20 (27.8%)	11 (14.7%)	9 (12.0%)
	61–70	56 (62.2%)	34 (37.8%)	18 (24.0%)	16 (21.3%)
	71+	23 (52.3%)	21 (47.7%)	11 (14.7%)	10 (13.3%)
	P	10.090		10.984	
Initial PSA (ng/ml)	2.5–4.0	19 (59.4%)	13 (40.6%)	11 (14.7%)	2 (2.7%)
	4.1–10.0	81 (65.3%)	43 (34.7%)	23 (30.7%)	20 (26.7%)
	>10.1	27 (64.3%)	15 (35.7%)	6 (8.0%)	9 (12.0%)
	p	10.830		0.013	
PSA Density	0–0.1	63 (73.3%)	23 (26.7%)	18 (24.0%)	5 (6.7%)
	0.11–0.2	48 (63.2%)	28 (36.8%)	14 (18.7%)	14 (18.7%)
	0.21+	20 (45.5%)	24 (54.5%)	8 (10.7%)	16 (21.3%)
	p	10.008*		10.008*	
PSA Change (ng/dl)	PSA ≤ 4.0 ng/dL	104 (64.6%)	57 (35.4%)	15 (20.0%)	3 (4.0%)
	PSA > 4.0 ng/dL or no change or increase	27 (60.0%)	18 (40.0%)	25 (33.3%)	32 (42.7%)
	P	10.571		10.003*	
PSA Change (%)	≥50% decrease	113 (61.7%)	70 (38.3%)	4 (5.3%)	1 (1.3%)
	<50% decrease or no change or increase	18 (78.3%)	5 (21.7%)	36 (48.0%)	34 (45.3%)
	P	10.121		20.364	
DRE	Unremarkable	120 (68.2%)	56 (31.8%)	33 (44.0%)	23 (30.7%)
	Suspicious	11 (36.7%)	19 (63.3%)	7 (9.3%)	12 (16.0%)
	P	10.001*		10.095	
PIRADS score on mpMRI	II	38 (86.4%)	6 (13.6%)	5 (83.3%)	1 (16.7%)
	III	22 (75.9%)	7 (24.1%)	6 (85.7%)	1 (14.3%)
	IV + V	20 (35.7%)	36 (64.3%)	16 (44.4%)	20 (55.6%)
	P	10.001*		n/a	

1 Chi-square test

2 Fisher’s exact test

* p < 0.05
